# The genetic diversity of triticale genotypes involved in Polish breeding programs

**DOI:** 10.1186/s40064-016-1997-8

**Published:** 2016-03-22

**Authors:** Agnieszka Niedziela, Renata Orłowska, Joanna Machczyńska, Piotr T. Bednarek

**Affiliations:** Department of Plant Physiology and Biochemistry, Plant Breeding and Acclimatization Institute - National Research Institute, 05-870 Radzików, Błonie, Poland

**Keywords:** Triticale, Genetic diversity, DArT markers

## Abstract

Genetic diversity analysis of triticale populations is useful for breeding programs, as it helps to select appropriate genetic material for classifying the parental lines, heterotic groups and predicting hybrid performance. In our study 232 breeding forms were analyzed using diversity arrays technology markers. Principal coordinate analysis followed by model-based Bayesian analysis of population structure revealed the presence of weak data structuring with three groups of data. In the first group, 17 spring and 17 winter forms were clustered. The second and the third groups were represented by 101 and 26 winter forms, respectively. Polymorphic information content values, as well as Shannon’s Information Index, were higher for the first (0.319) and second (0.309) than for third (0.234) group. AMOVA analysis demonstrated a higher level of within variation (86 %) than among populations (14 %). This study provides the basic information on the presence of structure within a genetic pool of triticale breeding forms.

**Electronic supplementary material:**

The online version of this article (doi:10.1186/s40064-016-1997-8) contains supplementary material, which is available to authorized users.

## Background

Triticale (X Triticosecale Wittmack) is a synthetic cereal crop that originated from a cross between *Triticum* species (AABB or AABBDD) and *Secale cereale* L. (RR). It combines grain quality and productivity typical for wheat with vigor, hardiness and high lysine content specific for rye (Myer and Barnett 2004). The vigorous root system and tolerance to abiotic stresses arising from rye (Niedziela et al. 2014) allow it to grow on light sandy soils with low fertility. Triticale has a broad range of applications. Its grain is mainly used for feeding and human consumption as well as for bread and food products (Peña 2004). Moreover, triticale is a potential energy crop, and putative source of biomass for bioethanol production (Wang et al. 1997).

The history of triticale started in 1875 with the first report describing a fertile hybrid between wheat and rye issued by the Scottish botanist Wilson (Wilson 1875). From this achievement to the first hexaploid triticales (Triticale No. 57 and Triticale No. 64) obtained by Hungarian breeder Kiss and released for commercial production passed almost 100 years (Kiss 1971). Substantial progress in triticale breeding was achieved by Tadeusz Wolski and his collaborators in 1968 (Ammar et al. 2004) who selected the first winter cultivar Lasko released in 1982. The variety became the widest grown triticale in the world. From 1987, Poland is the largest triticale maker with current production 4.3 mln tons of grain per year (http://www.factfish.com/catalog/crop).

Triticale hybrid breeding that started in the 1980s (Nalepa 1990) is one of the most promising directions in the species (Oettler et al. 2005) due to an efficient scheme and better control over variety distribution (Melchinger 1999). Separation of the maternal and paternal lines forms the basis of increased grain yields in hybrid breeding. Recently, cytoplasmic male sterility (CMS) system based on wheat *T. timopheevii* cytoplasm (Cauderon et al. 1985; Góral et al. 2006) replaced not allowed toxic chemical hybridization agents (Oettler et al. 2005). It was revealed that winter triticale hybrids with CMS Tt system showed 10–20 % relative midparent heterosis (MPH %) for grain yield (Oettler et al. 2005; Góral et al. 2006). The presence of sterilizing cytoplasm affects pollen grain development making maternal lines non-fertile. However, Tt cytoplasm is easily restored (Góral et al. 2010; Stojałowski et al. 2013) what makes the identification of effective maintainers and promising parental components for crosses that produce hybrids with superior yield problematic (Góral 2002; Warzecha et al. 2014). Currently, efforts are directed towards the understanding of the genetic background of male sterility in triticale. However, little is known about genes involved in the process except for the fact that the trait is multigenic, and each of the genes explains only a small part of the phenotypic variance (Stojałowski et al. 2013). Alternatively, genetic diversity within the breeding pool, analysis of genetic structure of the available materials as well as the knowledge of putative heterotic groups (Melchinger 1999; Fischer et al. 2010a, b) is of interest for breeders.

The evaluation of genetic diversity within the available genetic pool as well as the identification of the level of structure of the pool could be the easiest way to differentiate materials for breeding program purposes. Such data could be a prerequisite for the identification of putative maintainer or parental forms (Góral et al. 2005) or selection of components for crosses (Góral et al. 2006). Based on genetic distances evaluated using microsatellite markers (Tams et al. 2004; Kuleung et al. 2006; de Costa et al. 2007; Trebichalský et al. 2013) demonstrated that triticale germplasm is of high similarity. Likewise, Kuleung et al. (2006) divided 80 triticale worldwide accessions into five clusters with the average similarity equal to 0.45. Notable similarity (0.56) was also observed among Brazilian triticale forms (de Costa et al. 2007). Based on European accessions from 13 breeding companies 15.3 % of the variation among available materials was revealed (Tams et al. 2004) indicating the limited variability of the materials. Theoretically, the larger genetic distance between crossed forms the better performance of the offspring could be predicted (Moll et al. 1965). Nevertheless, there is evidence demonstrating that crossing even closely related lines may sometimes lead to reasonable grain yield (Fernandes et al. 2015). Thus, the exploitation of genetic variation and genetic structure may not necessary deliver the final solution for breeders but could be used as a preliminary recommendation for breeding programs. The other approach is based on the formation of heterotic groups where each group exhibits similar reaction (i.e. grain yield) with the same tester (Melchinger 1999; Fischer et al. 2010a). If combined with molecular data on diversity and population structure the evaluation of such groups could be of value for breeders (Melchinger 1999). Such an approach proved to work well in the case of rye where two heterotic groups were assessed (Fischer et al. 2010a). Their exploitation had an impact on hybrid rye breeding (Fischer et al. 2010a). However, in triticale, the evaluation of such groups is only at the very initial stage (Fischer et al. 2010a, b). Nevertheless, the assignment of the lines into heterotic groups increased the performance of the hybrid population by 2.8 % compared to control group where heterotic groups were not considered (Fischer et al. 2010a, b). Independently of whether genetic diversity, population structure or heterotic groups are studied the molecular characterization of plant materials is required what could be achieved using i.e. DArT platform (Alheit et al. 2011).

The objective of the study was to estimate genetic diversity and population structure of winter and spring triticale genotypes incorporated into Polish breeding programs using DArT markers for future triticale breeding programs.

## Results

In total 3117 DArTs polymorphisms were obtained based on 232 triticale breeding forms (193 winter and 39 spring genotypes). 1275, 1582 and 260 markers developed based on wheat, rye and triticale genomes, respectively were used. After the elimination of 1279 redundant and nine rare markers (identified in <5 % of cases), their respective numbers dropped to 1829 with 760, 912 and 157 in wheat, rye, and triticale sets. Among 232 genotypes, there were 22 spring and 49 winter forms having nearly identical counterparts that were substituted by their representatives. Finally, the study was conducted on a set of 161 diverse triticale forms (17 spring and 144 winters).

**Fig. 1:**
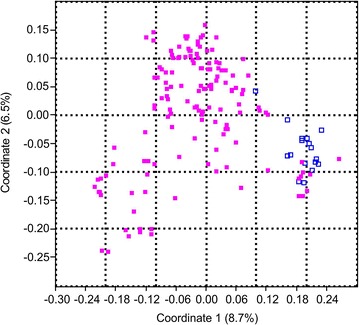
Principal coordinate analysis of the 161 genotypes based on Jaccard distances. *Percentages in parentheses* refer to the proportion of variance explained by the principal coordinate. *Violet filled square* states for winter forms; *Blue* ones reflect spring forms. Coordinate 1 explained 8.7 %, and Coordinate 2 explained 6.5 % of the total genetic variation

Based on principal coordinate analysis (PCoA) the first two coordinates explained the only small fraction of genetic variance (8.7 and 6.5 %, respectively). Nevertheless, three groups of data represented by most spring together with some winter forms plus two putative clusters of winter forms could be recognized (Fig. 1).

**Fig. 2:**
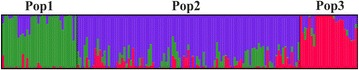
Graphical visualization of estimated population structure. *Plots* generated with the DISTRUCT software based on the Q-matrix consensus permuted across six replications for K = 3 using the CLUMPP software. *Vertical lines* demarcate the populations evaluated in Structure Harvester using Evanno method (Pop1, Pop2, and Pop3), with the widths of the corresponding *boxes* proportional to the numbers of individuals analyzed in the populations. The *coloring* indicates proportional cluster assignment for each individual

Population structure analysis followed by Evanno method (Evanno et al. 2005) recorded weak stratification (delta K = 58.4) with K equal to 3. Each specimen was assigned to one of the three groups (Fig. 2). In the first group (Pop1) all 17 spring forms together with 17 winter forms were clustered. The second (Pop2) and the third (Pop3) groups were represented by 101 and 26 winter forms, respectively.

The markers shared among structured genotypes were highly polymorphic for Pop1 (95.4 %) and Pop2 (97.3 %) (Table 1). Among them, 35 (Pop1) and 14 (Pop3) markers were unique for individual breeding forms. The lowest percentage of polymorphic loci was detected for Pop3 (75.6 %) with the absence of unique markers (Table 1).

**Table 1:** The arrangement of population genetic characteristics evaluated for the triticale populations identified via structure analysis

Population	Sample size	Type	P %	Unique markers	PIC ± SE	I ± SE
Pop1	34	Spring and winter	95.4	35	0.319 ± 0.004	0.478 ± 0.005
Pop2	101	Winter	97.3	14	0.309 ± 0.004	0.466 ± 0.005
Pop3	26	Winter	75.6	0	0.234 ± 0.004	0.356 ± 0.006
All (mean)	161	Spring and winter	89.5	–	0.287 ± 0.002	0.433 ± 0.003

P % states for the percentage of polymorphic loci, I reflects Shannon’s diversity index whereas PIC indicates polymorphic information content; *SE* standard error

The number of polymorphic markers shared by representatives of each group corresponded to 43.7, 40.7 and 30.7 % for Pop1, Pop2, and Pop3 respectively. Polymorphic information content (PIC) values varied among three groups of triticale forms (Table 1). Pop1 and Pop2 showed the highest PIC (0.319 and 0.309, respectively) whereas Pop3 the lowest (0.234). Shannon’s Information Index (I) matched PIC values and was the highest in Pop1 (0.478) and Pop2 (0.466) and the lowest in Pop3 (0.356).

AMOVA analysis of the model-based populations Pop1, Pop2, and Pop3 showed that 86 % of variance was due to within population whereas 14 % (p < 0.001) to among-population differences. Pairwise *Φ*_*PT*_ values indicated a high degree of differentiation between the populations Pop1 and Pop3 (0.219) and low genetic differentiation among populations Pop2 and populations Pop1 and Pop3 (0.109 and 0.138, respectively).

## Discussion

DArT is one of the marker systems adopted for the assessment of genetic diversity. The technology is considered to be efficient for getting accurate and reproducible markers, despite the low cost per data point (Jaccoud et al. 2001). However, the methodology favors the generation of redundant data due to the involvement of clones that may differ in size but have common sequences. Alternatively, markers having distinct sequences may have identical segregation patterns (Schouten et al. 2012; Raman et al. 2013). The presence of redundancy, as well as markers with low frequencies, may affect the statistical analysis and needs elimination. Up to 41 % of DArTs had to be removed from the analysis due to redundancy. The infrequent (rare) markers accounted for 0.3 % of data excluded from the analysis. Such a level of redundancy is in good agreement with the data presented in barley (38 %) (Wenzl et al. 2006) or *Arabidopsis thaliana* (43 %) (Wittenberg et al. 2005). Similarly, an amount of rare markers was also congruent with the results presented by the others (Wenzl et al. 2006). Insufficient information on the pedigree of the analyzed forms (as well as on their genetic diversity) resulted in the identification of similar or nearly identical ones that were preliminary included into the set. To avoid misleading population structuring, the final set of analyzed forms was reduced to 161 genetically distinct forms.

The studies on triticale diversity showed a vast worldwide similarity of the species (Furman et al. 1997; Tams et al. 2004; Alheit et al. 2011; Trebichalský et al. 2013). Such a situation is not surprising considering the way the species originated (Furman et al. 1997; Alheit et al. 2011). Both winter and spring triticale forms are closely related due to common breeding history and the presence of gene flow between both growth habits (Alheit et al. 2011). Breeders had frequently used lines from other programs as parents when developing breeding populations that resulted in a leaving out of the genetic diversity in triticale germplasm pool (Tams et al. 2004). Nevertheless, it does not lack diversity and genetic structure as indicated by data concerning European triticale (Fischer et al. 2010a, b). Our analysis demonstrated that the materials available for Polish breeders could be assigned to three groups represented by two clusters of winter forms and a group of materials encompassing winter and spring varieties as indicated by PCoA and structure analysis. However, the population structure was relatively weak indicating a limited level of differences among the groups.

It should be stressed that the analyzed spring forms were not distinguished from winter once based on PCoA and structure analysis. Lack of evident distinctiveness of spring and winter forms of triticale was demonstrated based on SSR markers (Kuleung et al. 2006). Moreover, Badea et al. (2011) and Alheit et al. (2011) found that the cultivar Matinal, classified as winter triticale, was close to the spring types. Such a grouping was explained by its pedigree that included a cross to the spring cultivar Colossal (Badea et al. 2011). The very similar explanation, at least partly, is applied in the case of our data demonstrating that five spring forms had a common winter ancestor (Additional file 1) (G. Budzianowski, personal communication, 2015). Moreover, the lack of apparent differentiation of the other spring and winter forms could be explained by common habit. In the case of Polish materials, the habitat for spring and winter forms is nearly the same. Clustering correlated with a winter or a spring habit of growth was revealed previously for triticale (Badea et al. 2011; Alheit et al. 2011), wheat (Chao et al. 2010), barley (Wang et al. 2012) and rapeseed (Bus et al. 2011). Alternatively, lack of apparent differentiation of spring and winter types could be related to the problems with marker system informativeness. However, the abovementioned explanation seems to be not the case as both polymorphic information context (PIC) as well as Shannon’s Information Indexes (I) evaluated based on DArT markers were relatively high with the highest PIC value above 0.3 for Pop1 and lowest (>0.2) for Pop3. Assuming that PIC value cannot exceed 0.5 (Alheit et al. 2011) in dominant markers, and that the higher values indicate greater informativeness of the marker system (Powell et al. 1996), our results stand this criterion. Nevertheless, the average PIC (0.287) was found to be small comparing to 0.36 (Badea et al. 2011) and 0.40 (Alheit et al. 2011) observed in studies of genetic diversity performed using DArT markers in triticale diverse worldwide accessions. However, taking into consideration the origin of the accessions (one breeding company) our results are in agreement with those of Bolibok-Bragoszewska et al. (2009) who observed lower PIC for DArTs in rye varieties from Polish breeding companies (0.27) than for wild accessions (0.39). Moreover, lower or similar PIC values for DArT markers were also observed i.e. in *Lesquerella* (0.21) (Cruz et al. 2013), *Asplenium* (0.21) (James et al. 2008), *Miscanthus* (0.22) (Tang et al. 2015), wheat (0.30) (Nielsen et al. 2014) and soybean (0.31) (Hahn and Würschum 2014), but still marker system was efficient for diversity studies.

Interestingly, DArT platform generated a high level of polymorphic markers shared by forms within each of the triticale populations evaluated via structure analysis. On the other hand, ANOVA showed that only 14 % of the variance was due to among-population differences. In that context, all our results demonstrate that the available variation among triticale forms is high enough, whereas structuring is due to a small fraction of them. Despite low value of among-population differences, which is comparable to wheat (12.41 and 15.6 %) (Mir et al. 2012; Hai et al. 2007) and barley (16.8 %) (Zhang et al. 2009), still there is enough within population variation for new varieties development. However, care needs to be taken to involve genetically diverse materials to avoid the erosion (Cowling 2013). Evidently, that seems to be the case in many Polish cultivars and breeding materials (Góral et al. 2005). Assuming the degree of diversity exhibited by Polish breeding materials comparable to that presented in European forms and the assessment of heterotic groups in that materials (Fischer et al. 2010a, b), it seems that such groups could also be elaborated for the Polish breeding pool. If heterotic groups are available one may expect an increase in grain yield up to 4 % as shown by the others (Fischer et al. 2010a, b).

## Conclusions

Triticale is one of the most successful cereals in Poland with new varieties entering the market each year (http://www.coboru.pl/polska/Rejestr/gat_w_rej.aspx). Unfortunately, knowledge covering genetic diversity, the population structure of gene pool and heterotic groups that constitute the base for lines, and hybrid breeding is limited; however, this could be easily achieved via marker analysis and may deliver background for the development of crosses in breeding programs.

Despite weak between population differences, the within-population variation in breeding triticale materials is high. Thus, the species preserves a relatively high level of variation to be exploited for breeding purposes; however, breeding programs need to be supported by molecular analysis to avoid diversity reduction. One of the interesting aspects of the presented data is the fact that breeding materials from only one company subdivided into three groups. Assuming that triticale programs carried by the other companies may use distinct genotypes one should expect further subclustering of the materials. The presence of the groups is the prerequisite for the identification of heterotic groups and exploitation of their potential in hybrid breeding programs.

## Methods

### Plant material and DArT genotyping

Two hundred and thirty-two triticale breeding forms kindly delivered by breeders (Strzelce Plant Breeders Ltd., Experimental Station Małyszyn, Poland) and consisted of 193 winters and 39 spring inbreed lines were used (the arrangement of the genotypes is given in Additional file 1).

The leaves were collected from single 14-day-old plant represented each line and homogenized in liquid nitrogen for DNA isolation. Total genomic DNA extraction was performed using Plant DNeasy MiniKit (Qiagen) followed by spectroscopic (NanoDrop ND-1000) measurement of its quantity. The DNA samples were subjected to 1.2 % agarose gel stained with ethidium bromide (0.1 μg/ml) in TBE buffer to verify DNA integrity.

The DArT marker analysis was provided by Triticarte Pty Ltd (Canberra, Australia; http://www.triticarte.com.au) using high-resolution triticale array (DArT) representing markers from wheat (wPt), rye (rPt), and triticale (tPt) according to the method described by Badea et al. (2011).

### Elimination of redundant data

For the identification of redundant markers, genetic distances among them were calculated according to Jaccard’s (1908) and clustered using the unweighted paired group method with an arithmetic mean (UPGMA) method. Computations were performed in PAST Software (Hammer et al. 2001). The robustness of each node of the dendrogram was estimated by 1000 bootstrap replications of the data (Nei and Kumar 2000). The markers were assumed to be identical if the differences between them did not exceed 5 %. The profiles of such markers were merged, and missing data were completed using information from the redundant markers of the contiguous assembly. Only one representative of the given redundant marker assembly was retained, and information on the removed markers was saved for further purposes. Rare alleles with low frequency (>95 %) along the individual profile were removed.

The data was also checked for the presence of identical or similar plant forms using agglomeration analysis (UPGMA) and Jaccard genetic distance in PAST software. The forms were assumed to be identical if the differences between them did not exceed 5 % and if their molecular profiles, except when missing markers, were identical. The profiles of such individuals were merged.

### Structure analysis

Preliminary visualization of putative structure among triticale breeding forms was calculated by the data from all non-redundant individuals and non-redundant markers. For this purpose, PCoA (Gower 1966) based on the Jaccard distances were performed with PAST software.

The population structure was evaluated using Bayesian analysis of the genetic structure (K) carried out with STRUCTURE 2.2.3 (Pritchard et al. 2000). Admixture model and allele frequencies correlated model were used. Each simulation was run using burn-in and MCMC (Markov Chain Monte Carlo) lengths of 100,000. The range of possible K was tested from 1 to 6. Six independent runs for each tested K were performed. The most likely number of K was evaluated following Evanno approach (Evanno et al. 2005) implemented in Structure Harvester software (Earl and von Holdt 2012). The averaged genetic structure was estimated in CLUMPP (Jakobsson and Rosenberg 2007). Six Q matrices were obtained in STRUCTURE for the given K. Graphical display of population structure was evaluated as an individual Q-matrix with Distruct1.1 software (Rosenberg 2004).

### Genetic diversity estimation

Basic indices of genetic diversity, including the percentages of polymorphic markers (P %), the number of unique markers, Shannon’s Diversity Index (I) (Shannon and Weaver 1949) were calculated in GenAlEx 5.3 EXCEL add-in (Peakal and Smouse 2001). Polymorphic information content (PIC) of dominant bi-allelic data was estimated by the formula: PIC = 1 − (p2  +  q2), where “p” is the frequency of present alleles and “q” is the frequency of null alleles (for diploid binary data and assuming Hardy–Weinberg Equilibrium, q = (1 − Band Freq.) * 0.5 and p = 1 − q) (Alheit et al. 2011).

Analysis of molecular variance—AMOVA (Φ_PT_ index values) was performed with GenAlex using 999 permutations test to estimate the reliability of the data.

## Additional file

10.1186/s40064-016-1997-8 Triticale winter (W) and spring (S) breeding forms.

## Abbreviations

AMOVA: analysis of molecular variance
DArT: diversity arrays technology
I: Shannon’s Information Indexes
PCoA: principal coordinate analysis
PIC: polymorphic information content
UPGMA: unweighted pair group method with arithmetic mean
