# Supplementary material for: The genetic diversity of triticale genotypes involved in Polish breeding programs
Source: Springerplus. 2016 Mar 22;5:355. doi: 10.1186/s40064-016-1997-8 (PMC4801839; doi:10.1186/s40064-016-1997-8)
Supplement: Supplementary file 1 — 10.1186/s40064-016-1997-8 Triticale winter (W) and spring (S) breeding forms. [file 40064_2016_1997_MOESM1_ESM.docx]

**Additional file 1**. Triticale winter (W) and spring (S) breeding forms.

| **Lp** | **Type (winter -W,**  **spring -S)** | **Form name** | **Pedigree of the form** |
| --- | --- | --- | --- |
| 1 | W | MAH 33427 [MZ-15] | MAH2797 x J-12 |
| 2 | W | PRESTO | DAGRO x LASKO |
| 3 | W | BOGO | MAH7810-501 x MAH 7686-2 |
| 4 | W | MARKO | ALMO x CHD762 |
| 5 | W | MAH 28047-3 | DN-14 x BOGO |
| 6 | W | 20 AMD | not available (ZSRR)* |
| 7 | W | DH 9/4/5 | not available (IHAR Radzików, Poland)* |
| 8 | W | MAH 29463-1 | [(ALMO x ARTHUR 71) x nn] x 1822/97 |
| 9 | W | MAH 31011-1 | Mo29688 x WOLTARIO |
| 10 | W | MAH 33116-2 | MAH3700 x DED251/98 |
| 11 | W | MAH 33259-2 | MAH27159-1 x LAD300/98 |
| 12 | W | MAH 32726-1/1 | MAH25311-1/1 x MAH2797 |
| 13 | W | MAH 32459-3 | ALZO x FDT901 |
| 14 | W | MAH 32722-8 | MAH25311-1/1 x KITARO |
| 15 | W | MAH 33056-1 | TORNADO x DED250 x 98 |
| 16 | W | PRESTO | DAGRO x LASKO |
| 17 | W | BOGO | MAH7810-501 x MAH 7686-2 |
| 18 | W | DH 9/4/5 | not available ( IHAR Radzików, Poland) |
| 19 | W | MAH 31938-2 | CFxxx x JANKO |
| 20 | W | MAH 31011-1 | Mo29688 x WOLTARIO |
| 21 | W | MAH 31331-1 | GUTEK x LAD447/94 |
| 22 | W | MAH 33116-2 | MAH3700 x DED251/98 |
| 23 | W | MAH 33116-2 | MAH3700 x DED251/98 |
| 24 | W | MAH 33259-1 | MAH27159-1 x LAD300/98 |
| 25 | W | MAH 31771-1 | [(Mo29611 x PANDA) x BOGO] x VALDY-3 |
| 26 | W | MAH 32459-3 | ALZO x FDT901 |
| 27 | W | MAH 33056-1 | TORNADO x DED250 x 98 |
| 28 | W | MAH 33264-2 | MAH27510-1 x DED251/98 |
| 29 | W | TITAN | not available (Romania)* |
| 30 | W | BOH 17-9 | not available (Borowo, Poland)* |
| 31 | W | BETA 2 | not available (Romania)* |
| 32 | W | CHD 1676/01 | not available (Choryń , Danko, Poland)* |
| 33 | W | MAH 28944-5 | MAH21899-1 x LAD1396 |
| 34 | W | MAH 31938-2 | CFxxx x JANKO |
| 35 | W | MAH 31938-1/1 | CFxxx x JANKO |
| 36 | W | MAH 29463-1 | [(ALMO x ARTHUR 71) x nn] x 1822/97 |
| 37 | W | MAH 32726-1 | (MAH25311-1/1 x MAH2797 |
| 38 | W | MAH 33259-2 | MAH27159-1 x LAD300/98 |
| 39 | W | MAH 33259-2 | MAH27159-1 x LAD300/98 |
| 40 | W | MAH 31771-1 | [(Mo29611 x PANDA) x BOGO] x VALDY-3 |
| 41 | W | MAH 33264-2 | MAH27510-1 x DED251/98 |
| 42 | W | MAH 33378 [MZ-9] | 1854 x MAH23217-2 |
| 43 | W | MAH 33424 [MZ-11] | MAH2797 x ALZO |
| 44 | W | MAH 33425 [MZ-14] | MAH2797 x 1851 |
| 45 | W | MAH 33425 [MZ-14] | MAH2797 x 1851 |
| 46 | W | MAH 33427 [MZ-15] | MAH2797 x J-12 |
| 47 | W | MAH 33443 [MZ-18] | MAH3198 x (MAH24507-2 x MORENO) |
| 48 | W | MAH 33467 [MZ-22] | MAH4202 x SZD456-7 |
| 49 | W | MAH 33474 [MZ-24] | ALIKO x 1852 |
| 50 | W | MAH 33494 [MZ-26] | MAH20112-1 x DED1231/97 |
| 51 | W | MAH 33544 [MZ-32] | MAH23175-1/20/24 x MAH25547-2 |
| 52 | W | MAH 33614 [MZ-33] | MAH26178-5 x UGO |
| 53 | W | MAH 33663 [MZ-35] | MAH247945-2 x MAH26371-2 |
| 54 | W | MAH 33663 [MZ-35] | MAH247945-2 x MAH26371-2 |
| 55 | W | MAH 33663 [MZ-36] | MAH247945-2 x MAH26371-2 |
| 56 | W | MAH 30854 [MZ-40] | (MAH1994 x PRESTO) x (MAH15238 x LAD785/87) |
| 57 | W | MAH 31011 [MZ-43] | Mo29688 x WOLTARIO |
| 58 | W | MAH 30577 [MZ-45] | [(Ugo x Roazon) x Ugo] x 1811/97 |
| 59 | W | MAH 31374 [MZ-46] | JANKO x PAWO |
| 60 | W | MAH 30035 [MZ-48] | WITON x 1811/97 (RHINO/spring x PRESTO/winter) |
| 61 | W | MAH 30035 [MZ-49] | WITON x 1811/97 (RHINO/spring x PRESTO/winter) |
| 62 | W | MAH 32459 [MZ-61] | ALZO x FDT901 |
| 63 | W | MAH 32476 [MZ-67] | DL-96-5-5 x ROTEGO |
| 64 | W | MAH 32476 [MZ-68] | DL-96-5-5 x ROTEGO |
| 65 | W | MAH 32720 [MZ-87] | [TORNADO x (ALMO x CHD762/85)] x KITARO |
| 66 | W | MAH 33072 [MZ-95] | WITON x DED251/98 |
| 67 | W | MAH 33084 [MZ-96] | MAH2797 x CHRONO |
| 68 | W | MAH 33113 [MZ-101] | TODAN x DED224/98 |
| 69 | W | MAH 33152 [MZ-115] | MAH4403 x MAH3700 |
| 70 | W | MAH 33152 [MZ-115] | MAH4403 x MAH3700 |
| 71 | W | MAH 33208 [MZ-119] | MAH25547-2 x DAD224/98 |
| 72 | W | MAH 32525 [MZ-125] | MAH2797 x KITARO |
| 73 | W | MAH 33264 [MZ-126] | MAH27510-1 x DED251/98 |
| 74 | W | MAH 33378 [MZ-9] | 1854 x MAH23217-2 |
| 75 | W | MAH 33424 [MZ-11] | MAH2797 x ALZO |
| 76 | W | MAH 33424 [MZ-11] | MAH2797 x ALZO |
| 77 | W | MAH 33443 [MZ-18] | MAH3198 x (MAH24507-2 x Moreno) |
| 78 | W | MAH 33467 [MZ-22] | MAH4202 x SZD456-7 |
| 79 | W | MAH 33474 [MZ-24] | ALIKO x 1852 |
| 80 | W | MAH 33474 [MZ-24] | ALIKO x 1852 |
| 81 | W | MAH 33474 [MZ-24] | ALIKO x 1852 |
| 82 | W | MAH 33494 [MZ-26] | MAH20112-1 x DED1231/97 |
| 83 | W | MAH 33494 [MZ-26] | MAH20112-1 x DED1231/97 |
| 84 | W | MAH 33614 [MZ-33] | MAH26178-5 x Ugo |
| 85 | W | MAH 31374 [MZ-46] | JANKO x PAWO |
| 86 | W | MAH 30035 [MZ-48] | WITON x 1811/97 (RHINO/spring x PRESTO/winter) |
| 87 | W | MAH 30035 [MZ-48] | WITON x 1811/97 (RHINO/spring x PRESTO/winter) |
| 88 | W | MAH 30035 [MZ-50] | WITON x 1811/97 (RHINO/spring x PRESTO/winter) |
| 89 | W | MAH 32476 [MZ-67] | DL-96-5-5 x ROTEGO |
| 90 | W | MAH 32476 [MZ-68] | DL-96-5-5 x ROTEGO |
| 91 | W | MAH 32476 [MZ-68] | DL-96-5-5 x ROTEGO |
| 92 | W | MAH 33072 [MZ-95] | WITON x DED251/98 |
| 93 | W | MAH 33084 [MZ-96] | MAH2797 x ChRONO |
| 94 | W | MAH 33113 [MZ-101] | TODAN x DED224/98 |
| 95 | W | MAH 33113 [MZ-101] | TODAN x DED224/98 |
| 96 | W | MAH 33424 [MZ-11] | MAH2797 x ALZO |
| 97 | W | MAH 33443 [MZ-18] | MAH3198 x (MAH24507-2 x MORENO) |
| 98 | W | MAH 33474 [MZ-24] | ALIKO x 1852 |
| 99 | W | MAH 33424 [MZ-11] | MAH2797 x ALZO |
| 100 | W | MAH 33424 [MZ-11] | MAH2797 x ALZO |
| 101 | W | MAH 33427 [MZ-15] | MAH2797 x J-12 |
| 102 | W | MAH 33427 [MZ-15] | MAH2797 x J-12 |
| 103 | W | MAH 33443 [MZ-18] | MAH3198 x (MAH24507-2 x MORENO) |
| 104 | W | MAH 33544 [MZ-32] | MAH23175-1/20/24 x MAH25547-2 |
| 105 | W | MAH 33663 [MZ-35] | MAH247945-2 x MAH26371-2 |
| 106 | W | MAH 33663 [MZ-35] | MAH247945-2 x MAH26371-2 |
| 107 | W | MAH 33663 [MZ-35] | MAH247945-2 x MAH26371-2 |
| 108 | W | MAH 33663 [MZ-36] | MAH247945-2 x MAH26371-2 |
| 109 | W | MAH 33663 [MZ-36] | MAH247945-2 x MAH26371-2 |
| 110 | W | MAH 33663 [MZ-36] | MAH247945-2 x MAH26371-2 |
| 111 | W | MAH 33663 [MZ-36] | MAH247945-2 x MAH26371-2 |
| 112 | W | MAH 29883 [MZ-37] | [(Mo6353-14 x PRESTO) x RAH101pH-4] x MAH2496 |
| 113 | W | MAH 29883 [MZ-37] | [(Mo6353-14 x PRESTO) x RAH101pH-4] x MAH2496 |
| 114 | W | MAH 29883 [MZ-38] | [(Mo6353-14 x PRESTO) x RAH101pH-4] x MAH2496 |
| 115 | W | MAH 30778 [MZ-39] | MAH2396 x KITARO |
| 116 | W | MAH 30854 [MZ-40] | (MAH1994 x PRESTO) x (MAH15238 x LAD785/87) |
| 117 | W | MAH 32475 [MZ-64] | DL-96-5-5 x BETA |
| 118 | W | MAH 32476 [MZ-67] | DL-96-5-5 x ROTEGO |
| 119 | W | MAH 32530 [MZ-73] | KRAKOWIAK x FDT901 |
| 120 | W | MAH 32720 [MZ-87] | [TORNADO x (ALMO x CHD762/85)] x KITARO |
| 121 | W | MAH 33098 [MZ-99] | TODAN x DED251/98 |
| 122 | W | MAH 33098 [MZ-99] | TODAN x DED251/98 |
| 123 | W | MAH 33098 [MZ-99] | TODAN x DED251/98 |
| 124 | W | MAH 33113 [MZ-101] | TODAN x DED224/98 |
| 125 | W | MAH 33113 [MZ-101] | TODAN x DED224/98 |
| 126 | W | MAH 33208 [MZ-119] | MAH25547-2 x DAD224/98 |
| 127 | W | MAH 33264 [MZ-126] | MAH27510-1 x DED251/98 |
| 128 | W | MAH 33264 [MZ-127] | MAH27510-1 x DED251/98 |
| 129 | W | DH 33370-2 | BOGO x MAH 24102-1/5 |
| 130 | W | DH 33370-3 | BOGO x MAH 24102-1/5 |
| 131 | W | DH 33434-1 | MAH3198 x DED 1231/97 |
| 132 | W | DH 33434-5 | MAH3198 x DED 1231/97 |
| 133 | W | DH 33434-7 | MAH3198 x DED 1231/97 |
| 134 | W | DH 33090-1 | MAH 3198 x CHD 2807/98-7-1 |
| 135 | W | DH 33192-1 | MAH 25163-2 x DED 224/98 |
| 136 | W | DH 33192-2 | MAH 25163-2 x DED 224/99 |
| 137 | W | DH 12932 | MAH 4705 |
| 138 | W | DH 33799-34 | B-123 USA x WITON |
| 139 | W | DH 33799-35 | B-123 USA x WITON |
| 140 | W | DH 33800-53 | B-123 USA x MAH 4302 |
| 141 | W | DH 34085-62 | MAH 3098 x DH 265-1 |
| 142 | W | DH 34106-34 | MAH 4604 x DED 251/98 |
| 143 | W | DH 34254-10 | MAH 28853-3/1 x DED 2175 |
| 144 | W | DH 34123-1 | N7153 x MODERATO |
| 145 | S | MAH 31577-1 | 6949/97 x CHD-93/96 |
| 146 | S | MAH 32388-4 | 6995/01 x KARGO |
| 147 | S | MAH 32388-8 | 6995/01 x KARGO |
| 148 | S | MAH 32392-2 | 7000/01 x MIGO |
| 149 | S | MAH 32928-24 | MAH 2500 x ALTER |
| 150 | S | MAH 33440-1 | MATEJKO x CHD 400 |
| 151 | S | ITYN 21 | HARE 132 x TESMO 3) x ZEBRA 79 , (CIMMYT) |
| 152 | S | ITYN 23 | KER 1, (CIMMYT) |
| 153 | S | ITYN 29 | PANCHE 424 x YOGUI 1, (CIMMYT) |
| 154 | S | ITYN 31 | TESMO 1 / MUS 603, (CIMMYT) |
| 155 | S | MAH 28293-1/14 | (MAJA x ETA) x GABO |
| 156 | S | DH 33486-3 | MAH 32969-15 x DUBLET |
| 157 | S | DH 33505-3 | MAH 3405(MILEWO) x MATEJKO |
| 158 | S | MAH 32975-2 | MAH 27470-1 x BACUM |
| 159 | S | MAH 32975-2 | MAH 27470-1 x BACUM |
| 160 | S | DUBLET | TJ 28/90 x TJ 53/90 |
| 161 | S | MAH 32948-1 | MAH 2601(MATEJKO) x CARACAL |

* not available
